# On the Inside October 2021

**DOI:** 10.1093/plphys/kiab378

**Published:** 2021-10-04

**Authors:** Peter V. Minorsky

**Affiliations:** School of Health and Natural Sciences, Mercy College, Dobbs Ferry, New York, NY, USA

## TAG remodeling to accumulate industrial oils

Hydroxy fatty acids (HFAs) are used in a wide variety of industrial products, including lubricants, biodegradable polyesters, and biofuels. The major source of HFAs is castor (*Ricinus communis*) oil that contains the 18-carbon HFA ricinoleic acid. Since castor seeds also produce the extremely toxic protein ricin, efforts are underway to engineer valuable HFAs into more suitable crops. However, with few exceptions, the bioengineering of unusual fatty acid production into model and crop plants to date has failed to achieve levels near those of native species. Oilseed plants accumulate triacylglycerol (TAG) up to 80% of seed weight: the TAG fatty acid composition largely determines its nutritional or industrial value. The inefficient flux of transgenic fatty acids through membrane lipids prior to incorporation into TAG has been characterized as a major bottleneck to oilseed engineering. An alternative strategy that bypasses this bottleneck is the remodeling of pre-existing TAG. Bhandari and Bates (pp. 799–815) report that such TAG remodeling mechanisms seem to be at work in developing *Physaria fendleri* seeds, that have been shown to accumulate TAG containing >60% lesquerolic acid, an unusual 20 carbon HFA that accumulates at only the *sn-1* and* sn-3* positions of TAG. Isotopic tracing of developing *P. fendleri* seed lipid metabolism identified that phosphatidylcholine-derived diacylglycerol is utilized to initially produce TAG with only one lesquerolic acid. Subsequently, a nonhydroxylated fatty acid is removed from TAG (transiently reproducing diacylgylcerol) and a second lesquerolic acid is incorporated. Thus, a dynamic TAG remodeling process involving anabolic and catabolic reactions controls the final TAG fatty acid composition. The authors’ reinterpretation of *P. fendleri* transcriptomic data identified potential genes involved in TAG remodeling that could provide a new approach for oilseed engineering by altering the fatty acid composition of oils after initial TAG synthesis.

## sRNA regulation of two Calvin–Benson cycle enzymes

In response to nitrogen (N) deficiency, some filamentous cyanobacteria form a heterocyst, a specialized cell type devoted to the fixation of atmospheric N_2_. The differentiation of functional heterocysts involves a precise gene expression program in which many genes are distinctly regulated in cells undergoing the transformation. This differentiation process is controlled by the transcriptional regulator NtcA. The activation of NTcA leads to extensive metabolic reprogramming, including the shutdown of photosynthetic CO_2_ fixation in heterocysts: this helps to provide a microaerobic environment suitable for N_2_ fixation. However, post-transcriptional regulatory mechanisms might also play a relevant role in achieving properly balanced assimilation of carbon and N. Small regulatory RNAs (sRNAs) are emerging as major post-transcriptional regulators of gene expression. Brenes-Álvarez et al. (pp. 787–798) now report the participation of an sRNA called nitrogen stress-inducible RNA 4 (NsiR4) in the post-transcriptional regulation expression of two genes involved in CO_2_ fixation via the Calvin cycle, namely *glpX*, which encodes sedoheptulose-1,7-bisphosphatase/fructose-1,6-bisphosphatase (SBPase), and *pgk*, which encodes phosphoglycerate kinase (PGK). The overexpression of NsiR4 in the cyanobacterium *Nostoc* resulted in a reduced amount of SBPase protein and reduced PGK activity, as well as reduced levels of both *glpX* and *pgk* mRNAs. NsiR4 is also more strongly expressed in heterocysts than in vegetative cells, which may contribute to the heterocyst-specific shutdown of Calvin cycle flux. Post-transcriptional regulation of two Calvin cycle enzymes by NsiR4, an N-regulated sRNA, represents an additional link between N control and CO_2_ assimilation.

## Nanoparticle delivery of dsRNA into tomato pollen

Transgenes expressing double-stranded RNA (dsRNA) have been particularly effective in conferring resistance to viruses and pests, and in manipulating metabolic pathways in plants. However, the efficacy of genetic engineering and virus-facilitated trait modification varies greatly between different plant species. In the past decade, many nanoparticles have been developed to mediate delivery of biomolecules into plants. These include single-walled carbon nanotubes that can deliver plasmids and siRNAs into leaves via infiltration. Nanoparticle delivery platforms have many advantages, such as low toxicity, high delivery efficiency, species independence, and subcellular targeting. Despite their potential, however, there have been few reports of nanoparticle-mediated delivery of functional biomolecules directly into intact plant cells. Layered double hydroxide (LDH) is a family of bio-compatible and degradable clay materials. Plate-like LDH nanoparticles consist of mixed divalent/trivalent metal hydroxide layers, which are bonded together with anions and water molecules between them. The layered structure and positively charged hydroxide layers make the LDH nanoparticle a suitable carrier to deliver negatively charged biomolecules such as DNA, RNA, and proteins. Yong et al. (pp. 886–899) show that LDH nanoparticles are taken up by developing pollen. Using *β-glucuronidase* (*GUS*) transgene as the reporter, they found that LDH-delivered functional dsRNA efficiently induces RNAi of the *GUS* gene and reduces the GUS enzyme activity. These findings demonstrate that an LDH delivery platform with the size of up to 50 nm in diameter can facilitate delivery of functional exogenous dsRNA into intact plant cells.

## Evaluation of RALF family peptides

RAPID ALKALINIZATION FACTOR (RALF) peptides belong to a family of cysteine-rich plant peptide hormones that are involved in multiple physiological and developmental processes, ranging from pollen tube growth to the modulation of immune responses. They were originally discovered in a peptide hormone screen due to their ability to cause medium alkalinization of cell cultures. Later, RALF peptides with several conserved motifs were found to be ubiquitous in terrestrial plants. In Arabidopsis (*Arabidopsis thaliana*), more than 30 RALF peptides have been predicted. Nevertheless, to date, there is no comprehensive, family-wide functional study of RALF peptides. Abarca et al. (pp. 996–1010) have analyzed the phylogeny of the proposed multigenic RALF peptide family in the Arabidopsis ecotype Col-0, and tested a variety of physiological responses triggered by RALFs. They show that the majority of AtRALF peptides, when applied exogenously as synthetic peptides, induce seedling or root growth inhibition and modulate ROS production in Arabidopsis. These findings suggest that alkalinization and growth inhibition are, generally, coupled characteristics of RALF peptides. Finally, the authors provide evidence that for the majority of these peptides, their responses are dependent on the receptor kinase FERONIA, suggesting a pivotal role for this receptor in the perception of multiple RALF peptides.

## Impact of respiration on photosynthetic activity

Respiration in photosynthetic organisms is essential for supporting energy demand in all plant tissues at night, in nonphotosynthetic tissues such as roots, and during developmental stages where photosynthesis is not active (e.g. seed germination). Increasing evidence, however, suggests that respiration is also important for sustaining optimal photosynthetic activity, and that there are strong functional links between the bioenergetics of chloroplasts and mitochondria. To further elucidate these links, Mellon et al. (pp. 931–946) isolated *Physcomitrella* (*Physcomitrium patens*) mutants with altered respiration stemming from the inactivation of Complex I (CI) of the mitochondrial electron transport chain. Inactivation of CI caused a strong impairment of growth even in fully autotrophic conditions in tissues where all the cells are photosynthetically active. The impact of CI mutations on respiratory activity was assessed by measuring oxygen (O_2_) consumption rate. In CI mutants, O_2_ consumption in the dark was higher than in wild-type (WT). While this seems counterintuitive for mutants affected in a respiratory complex, this observation can be explained by the compensatory over-accumulation of Complexes II and II and alternative terminal oxidase. While CI mutants showed alterations in the stoichiometry of these respiratory complexes, the composition of the photosynthetic apparatus was not substantially affected. Photosynthetic functionality is nevertheless altered in CI mutants, and plants exposed to sub-saturating illumination show several differences. Both PSI and PSII showed a higher yield upon illumination in mutant plants, suggesting a lower saturation level in illuminated plants. CI mutants also show increased proton conductivity across the thylakoids membrane that, combined with an equivalent proton motive force, is consistent with a higher electron transport rate. The higher ATPase activity in the CI mutants causes a faster translocation of protons in the stroma and an increase in the lumenal pH, in turn, affecting photosynthesis regulatory mechanisms modulated by lumen acidification. These results demonstrate that alteration of respiratory activity directly impacts photosynthesis in *P. patens* and that metabolic interaction between organelles is essential in their ability to use light energy for growth.

## Root hairs and water uptake under water deficit

Although the role of root hairs in nutrient acquisition has been well documented, their contribution to water uptake remains controversial. Many of these studies have involved comparisons of hairless mutants to WTs, but the general conclusions drawn have often been contradictory. Cai et al. (pp. 858–872) hypothesize that the differences in the interactions between root hairs and soils of different textures and hydraulic properties may reconcile many of these seeming discrepancies. They hypothesize: (1) root hairs attenuate the drop in soil matric potential around the roots, by extending the surface of the roots extracting water and thus reducing the flow velocity at the root–soil interface. This effect is expected to be particularly marked in coarse-textured soils, such as sandy soils, whose hydraulic conductivity abruptly drops with decreasing soil matric potentials; (2) the effect of root hairs on the relation between transpiration rate and leaf water potential is more pronounced in sand than in loam, due to the pronounced drop in soil hydraulic conductivity of sand; (3) mutant plants develop longer and thicker roots to partly compensate for the absence of root hairs. To test these hypotheses, they compared the maize (*Zea mays*) mutant (*roothairless*, *rth3*) versus WT. These two genotypes were grown in two contrasting soil textures: sand and loam. Transpiration rate and leaf water potential were measured continuously for different soil moisture conditions using the root pressure chamber method. Based on their findings, the authors propose a soil–plant hydraulic model to explain the relationship between transpiration rate and leaf xylem water potential. They used this model to systematically compare the two genotypes in the two soil textures and to investigate whether, thanks to root hairs, the two genotypes would exhibit distinct root hydraulic properties. Contrary to previous observations with barley, they found no significant effect of root hairs on water uptake and soil–plant hydraulic conductance in both sandy and loamy soils.

